# Supplementary material for: Usp9x regulates Ets-1 ubiquitination and stability to control NRAS expression and tumorigenicity in melanoma
Source: Nat Commun. 2017 Feb 15;8:14449. doi: 10.1038/ncomms14449 (PMC5316860; doi:10.1038/ncomms14449)
Supplement: Supplementary Information — Supplementary Figures, Supplementary Tables and Supplementary References. [file ncomms14449-s1.pdf]

## Supplementary Information

***Supplementary Fig. 1. Elevated Usp9x in melanoma and NRAS mutant melanoma cells are dependent on NRAS for 3D growth.***

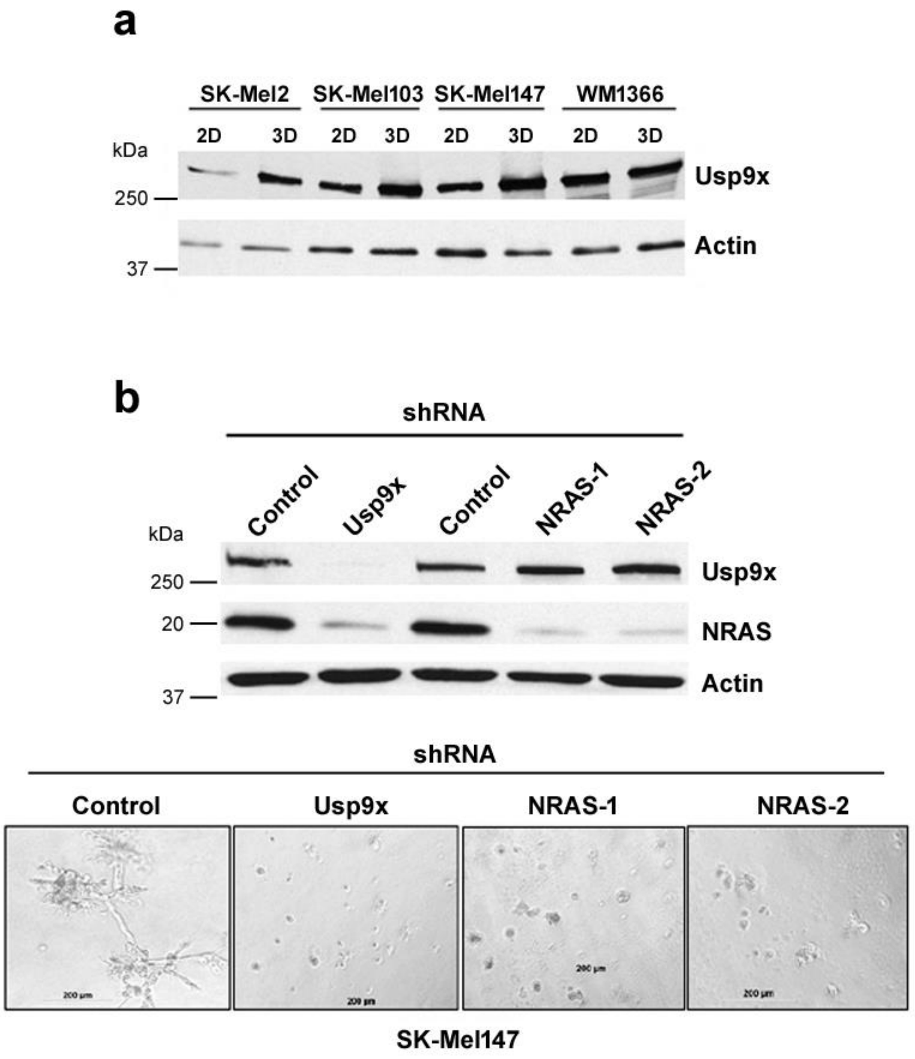


**a**. Immunoblot for Usp9x protein in NRAS mutant melanoma cells grown in 2D (monolayer) or 3D (agarose) for 3 days. Actin served as a loading control. **b.** Top - Immunoblot for Usp9x and NRAS protein in SK-Mel147 melanoma cells with shRNA-mediated Usp9x or NRAS (NRAS-1 and NRAS-2) KD. Bottom - Colony formation and expansion in control, Usp9x KD or NRAS KD SK-Mel147 cells (200 cells plated) grown on matrigel for 7 days.

*Supplementary Fig. 2. Identifying proteins deubiquitinated by Usp9x.*

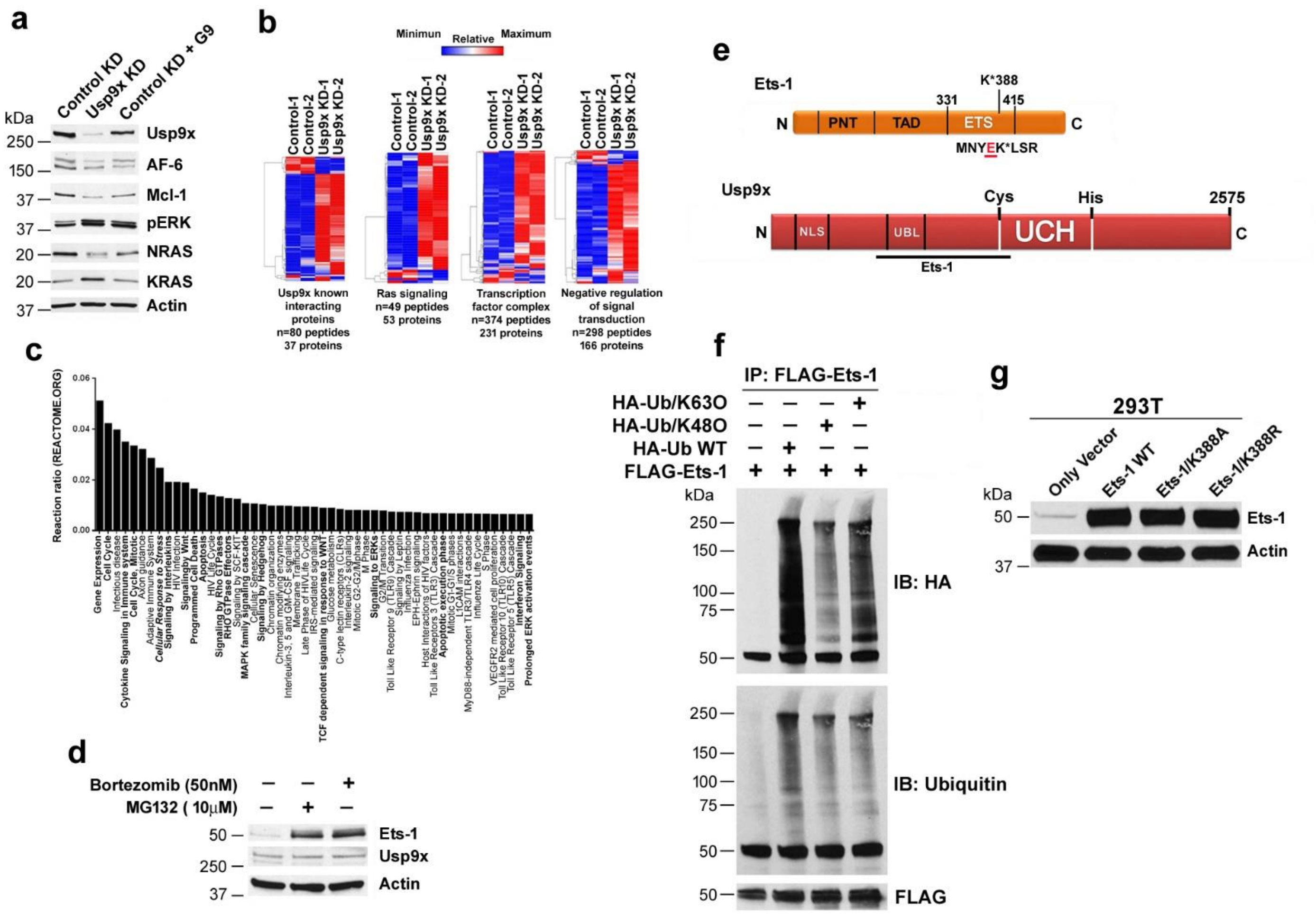


**a.** Immunoblot for known substrates of Usp9x in NRAS mutant SK-Mel147 melanoma cells after treatment as indicated. Lysates from this assessment were subjected to UbiScan (Cell Signaling Technologies, Inc.) analysis. **b**. Heat map analysis of ubiquitination changes in known Usp9x-interacting proteins (total=104; PubMed, GENE) following Usp9x KD in melanoma cells (37 proteins showed altered ubiquitination). Heat maps of differentially ubiquitinated proteins involved in RAS signaling, transcription factor complexes and negative regulation of signal transduction. Heat maps represent peptides with a coefficient of variation (CV) > 50. Complete protein list provided in the Supplemental Data files 1-7. The number of unique peptides and proteins reproducibly detected in each analysis is shown. **c.** Reactome (molecular pathways) analysis of proteins differentially ubiquitinated after Usp9x KD in NRAS mutant melanoma cells (Supplementary Data file 8). Enriched groups are ranked by the most significant reactome ratio. **d**. SK-Mel147 cells were treated as described for 8 h before assessing Ets-1, Usp9x and actin levels by immunoblotting. **e.** Schematic diagram of the human Ets-1 protein with specific domains and predicted site of Usp9x deubiquitination (K*388). Usp9x (full length 2575 aa) protein showing the location of the NLS (nuclear localization sequence), UBL (ubiquitin-like domain), UCH (ubiquitin C-terminal hydrolase domain) and predicted site of Ets-1 interaction. **f**. K63-linked ubiquitination of Ets-1. HEK293T cells were co-transfected with FLAG-Ets-1 (WT) and pRK5-HA-ubiquitin (WT), pRK5-HA-Ub/K48 only and pRK5-HA-Ub/K63 only expression constructs. After 48 h, FLAG-Ets-1 was immunoprecipitated and immunoblotted with antibodies against HA and Ubiquitin to detect ubiquitinated Ets-1. **g.** HEK293T cells were transfected with an expression vector for HA-Ets-1 WT or specific K388 mutants (as indicated). After 48 h, cell lysates were immunoblotted for HA (Ets-1). Actin served as a protein loading control.

*Supplementary Fig. 3. Usp9x controls Ets-1 and NRAS protein levels.*

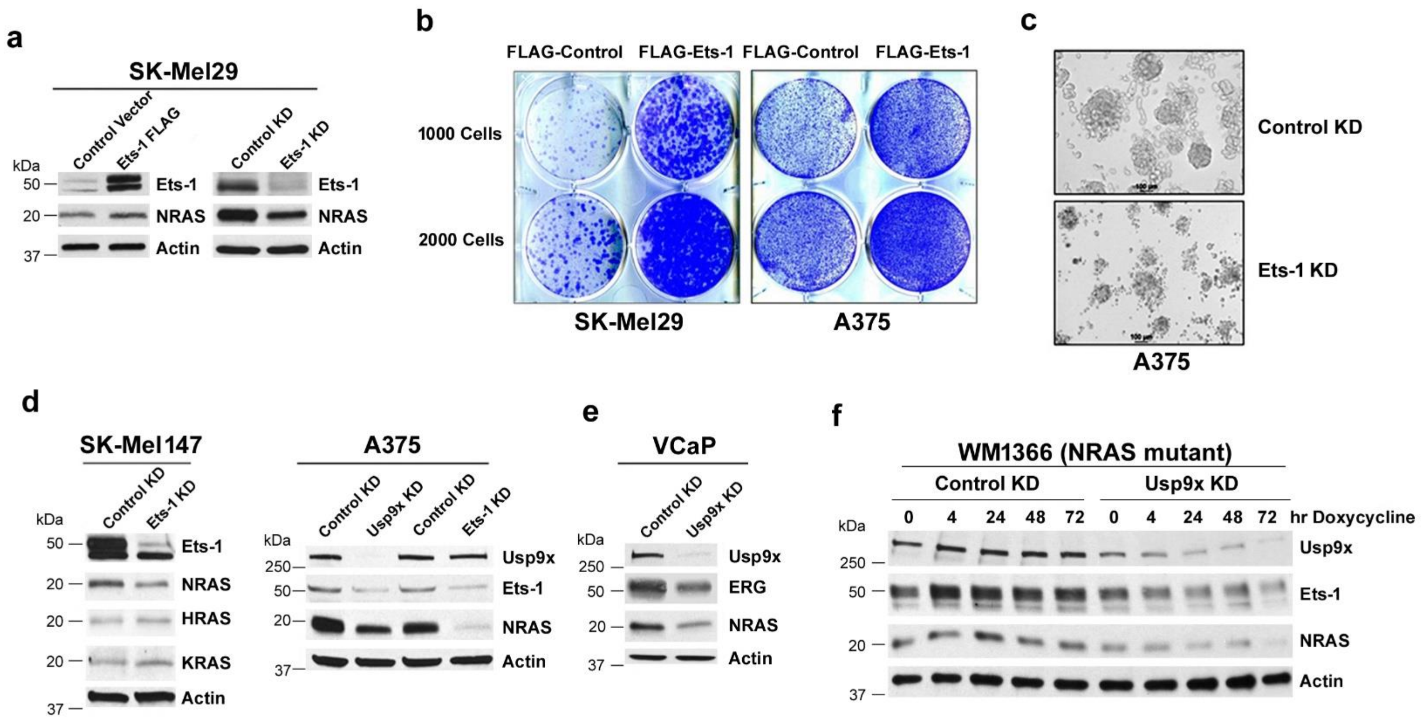


**a**. Immunoblot for Ets-1 and NRAS in BRAF mutant SK-Mel29 cells with Ets-1-overexpression (Ets-1 FLAG; left) or KD (right). **b**. Crystal violet staining of FLAG-Control or FLAG-Ets-1 SK-Mel29 (left) or A375 (right) cells grown for 3 weeks in 6-well plates. **c**. Phase contrast images of control and Ets-1 KD BRAF mutant A375 cells grown on matrigel (3D) for 7 days (black bar represents 100 μm). **d.** Immunoblot for Ets-1, NRAS, HRAS and KRAS protein in control and Ets-1 KD SK-Mel147 NRAS mutant melanoma cells after 5 days of KD (left); immunoblot for Ets-1, Usp9x, NRAS and actin in control, Usp9x KD and Ets-1 KD BRAF mutant A375 melanoma cells after 5 days of KD (right). **e**. Immunoblot assessment of Usp9x, ERG, NRAS and actin protein levels in control and Usp9x KD VCaP ERG positive prostate cancer cells. **f**. Immunoblot for the protein indicated in lysates from WM1366 cells subjected to infection, selection and induction of shRNA (as indicated) with doxycycline-inducible vectors (TRIPZ). Lysates derived from cells treated with doxycycline (1 μg/ml) for the interval indicated are shown.

*Supplementary Fig. 4. Usp9x controls Dusp4 protein levels.*

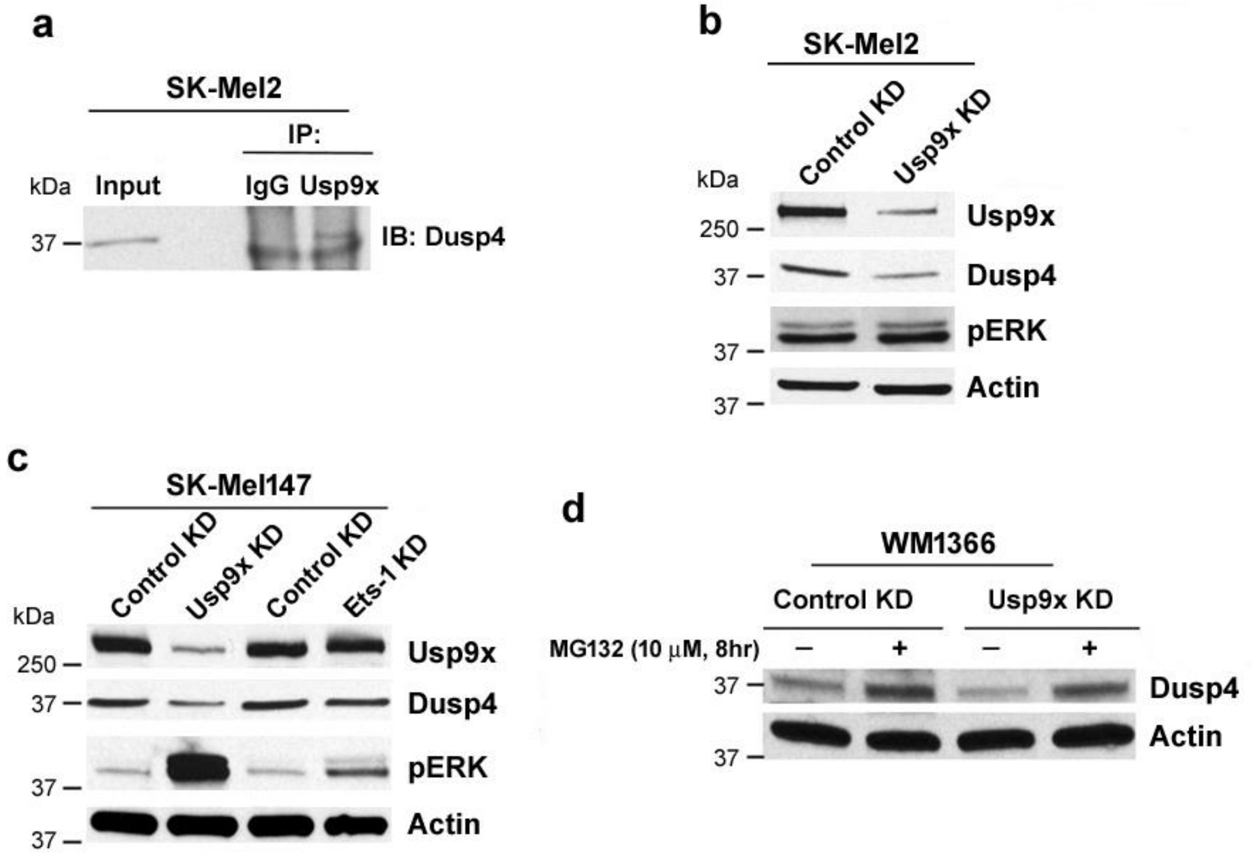


**a.** Anti-Usp9x or control IgG pulldowns from NRAS mutant SK-Mel2 cells were immunoblotted for Dusp4 and compared to Dusp4 levels in total cell lysates (input) (control IgG band is light chain Ig). **b.** Immunoblot for Usp9x, Dusp4, pERK and actin in control and Usp9x KD NRAS mutant SK-Mel2 melanoma cells after 5 days of KD. **c.** Immunoblot for Usp9x, Dusp4, pERK and actin in SK-Mel147 cells after Usp9x or Ets-1 KD. **d**. Immunoblot for Dusp4 and actin in Usp9x KD NRAS mutant melanoma WM1366 cells treated with MG132 for 8 h.

***Supplementary Fig. 5. Primary and metastatic melanoma tumors express high levels of Usp9x and Ets-1.***

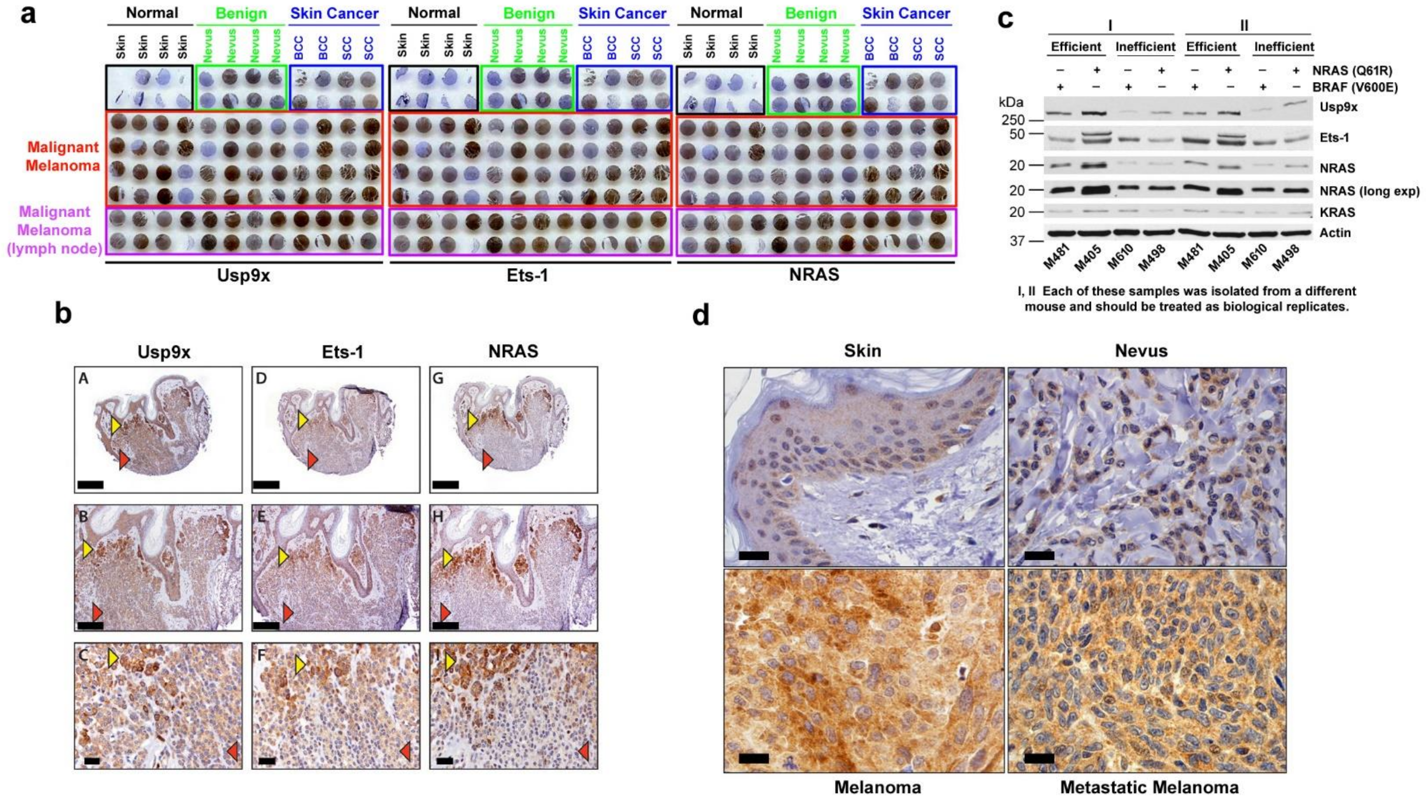


**a**. Immunostaining for Usp9x, Ets-1 and NRAS protein expression in a tissue microarray of normal skin, benign nevus, basal cell carcinoma, squamous cell carcinoma, malignant melanoma and metastatic melanoma (described in Figure 5). **b**. Stratified expression of Usp9x, Ets-1 and NRAS with dermal maturation in a benign nevus. The superficial aspect of the nevus (yellow arrowheads) demonstrates robust expression of all three proteins, which is diminished in the deeper aspect of the nevus (red arrowheads). The black bar represents 20 μm. **c.** Tumor tissue from primary human melanoma explants established in NSG mice [1] was assessed by immunoblot for Usp9x, Ets-1, NRAS (short and long exposure) and KRAS protein expression (NRAS and BRAF mutation status verified by the University of Texas Southwestern Medical Center, Dallas, TX). **d.** Immunostaining for Ets-1 in normal skin, benign nevus, primary melanoma and metastatic melanoma (from Fig. 5h) photographed under high magnification (1000X). Black bars represent 20 μm.

***Supplementary Fig. 6. ChIP-SEQ analysis of Ets-1 occupancy near the NRAS gene.***

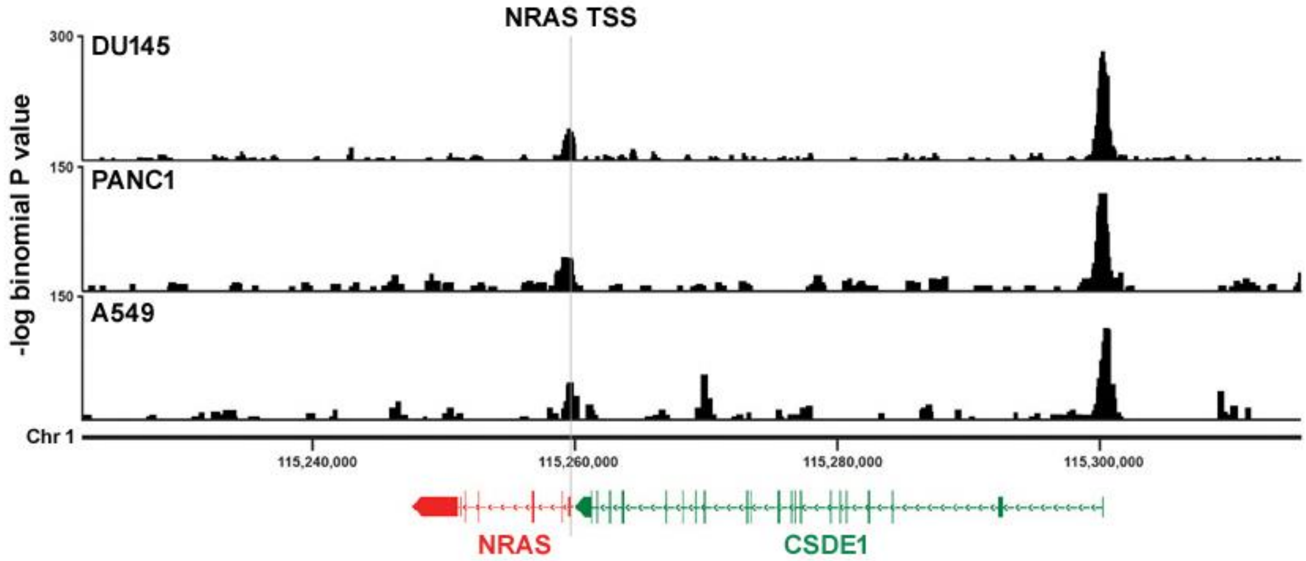


Ets-1 occupancy near the gene encoding NRAS is plotted by -log binomial *P*-value from ChIP-SEQ data from the prostate (DU145), pancreatic (PANC1), and lung (A549) cell lines. A549 data is from ENCODE (Richard Myers Group, GSM1010829).

***Supplementary Fig. 7. Ets-1 is induced by BRAF and MEK kinase inhibition.***

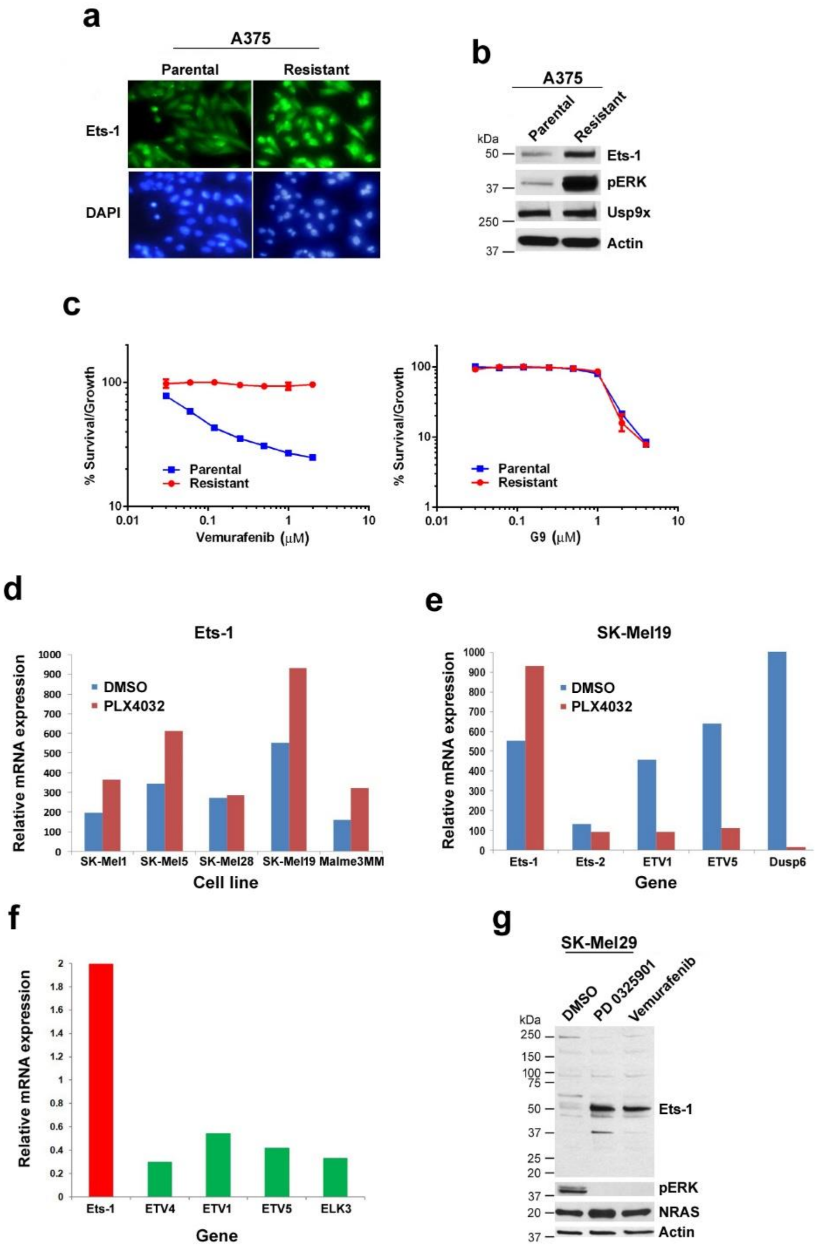


**a.** Parental and vemurafenib-resistant A375 melanoma cells [2] were plated on glass cover slips, fixed and stained for Ets-1 protein and detected with fluorescently tagged anti-IgG. DAPI staining was used to detect cell nuclei. **b**. Parental and vemurafenib-resistant A375 cells were immunoblotted for the proteins indicated. **c.** Cells from **b** plated in individual wells of a 96-well plate were treated with the indicated dose of compound for 72 h before cell proliferation was assessed by MTT assay. The results represent the average +/- S.D. of 4 replicates. **d**. Ets-1 expression (taken from a microarray probe set, accession no. GSE20051) in BRAF-mutant melanoma cell lines treated with BRAF inhibitor (250 nM PLX4032, 8 h), as previously described [3]. Seventy-three genes were reported to be altered by BRAF inhibition in this microarray. **e**. ETS family member expression in the same microarray described in d. Relative levels are compared to untreated controls. **f**. ETS family member expression (taken from a microarray; GO: transcription factor activity: 00037000) in control and BRAF inhibitor (0.1 μM for 6 hr) treated A375 cells, as recently described [4]. **g**. Total immunoblot for Ets-1, pERK, NRAS and actin in BRAF mutant SK-Mel29 cells treated with Vemurafenib (1 μM) or PD0325901 (0.5 μM) for 24 h.

Supplementary Figure 8 - Representative uncropped Western blots corresponding to the figure designated.

## Figure 1

**a**

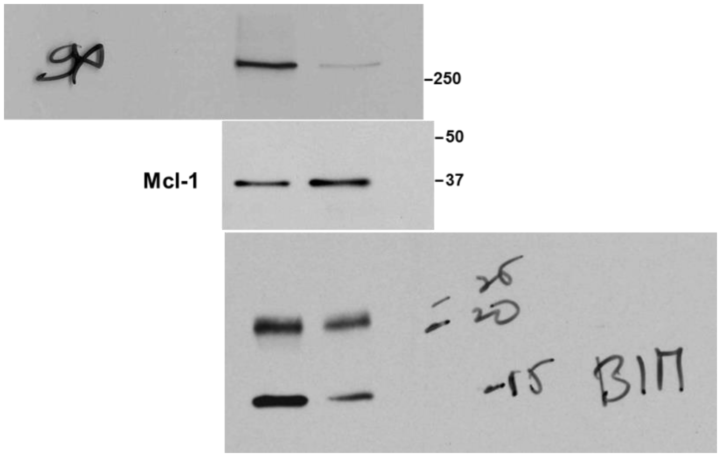


**f**

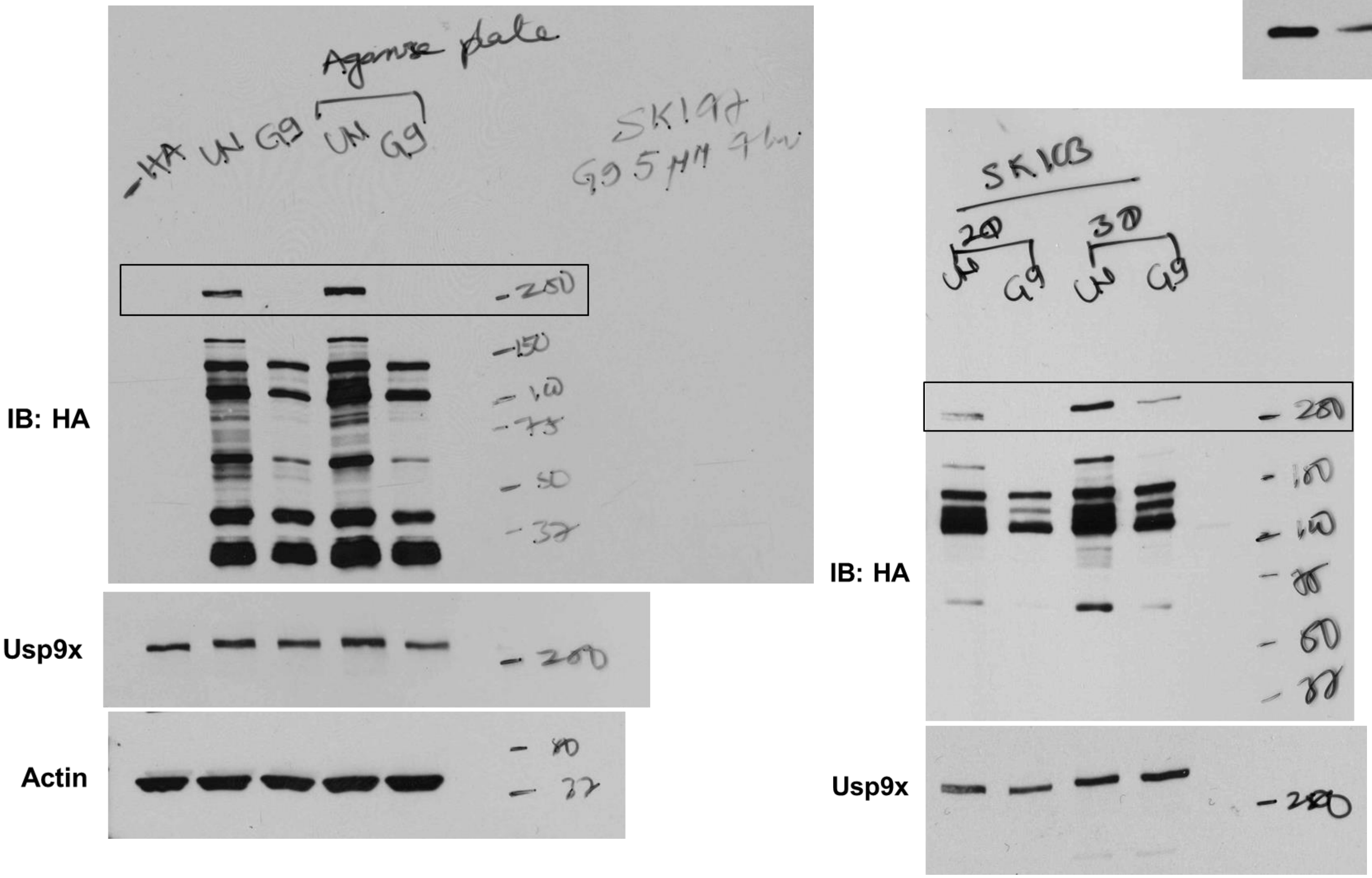

# Figure 2

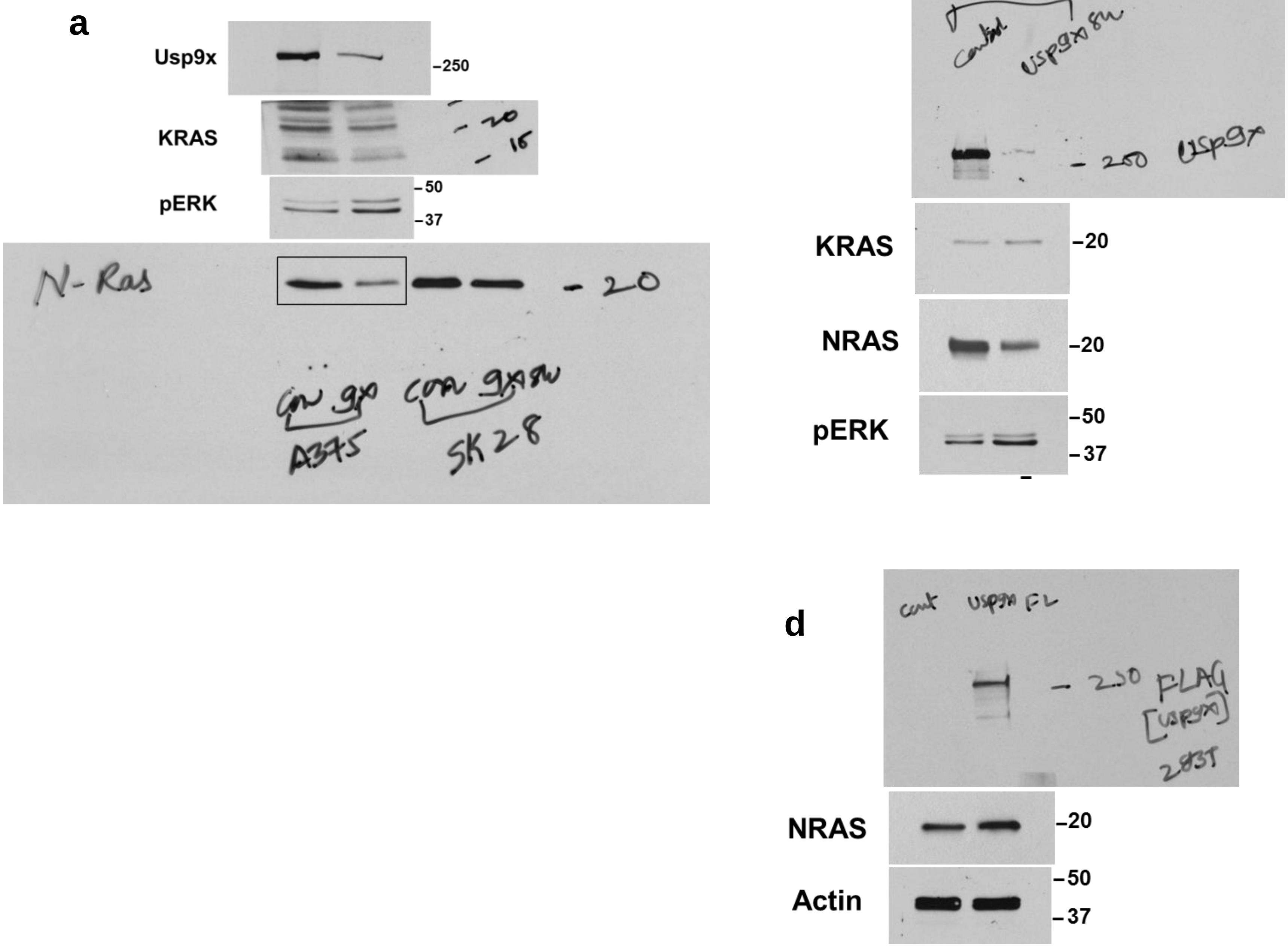
a
Usp9x
–250
KRAS
pERK
–50
–37
N-Ras
– 20
A375
SK28
SK-147
Control
– 250
KRAS
–20
NRAS
–20
pERK
–50
–37
d
cont
– 250 FLAG
293T
NRAS
–20
Actin
–50
–37

Figure 3

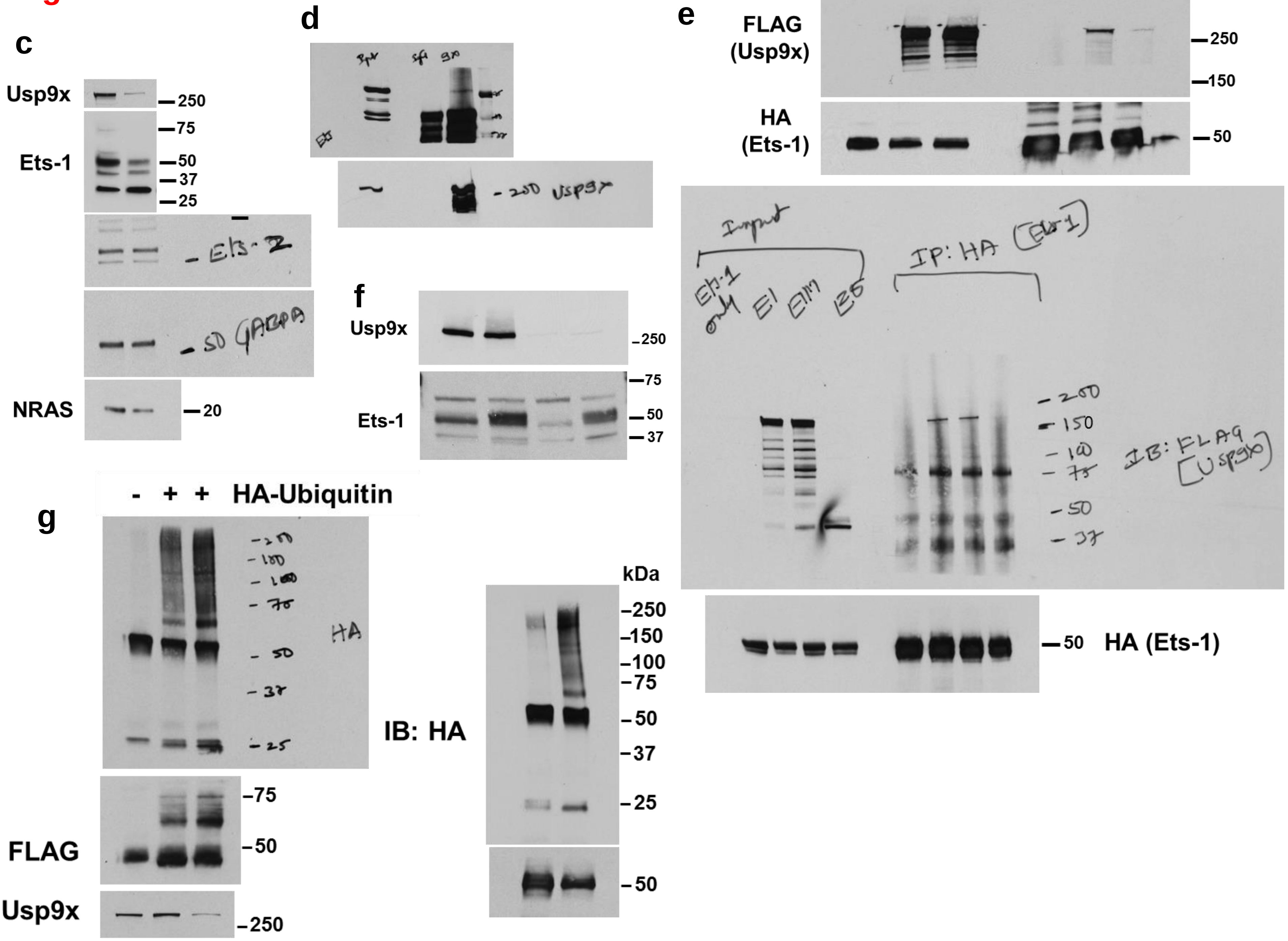

c
Usp9x
250
75
Ets-1
50
37
25
NRAS
20
d
e
FLAG (Usp9x)
250
150
HA (Ets-1)
50
f
Usp9x
250
Ets-1
75
50
37
HA (Ets-1)
g
- + + HA-Ubiquitin
IB: HA
kDa
250
150
100
75
50
37
25
FLAG
75
50
Usp9x
250

**Figure 4**

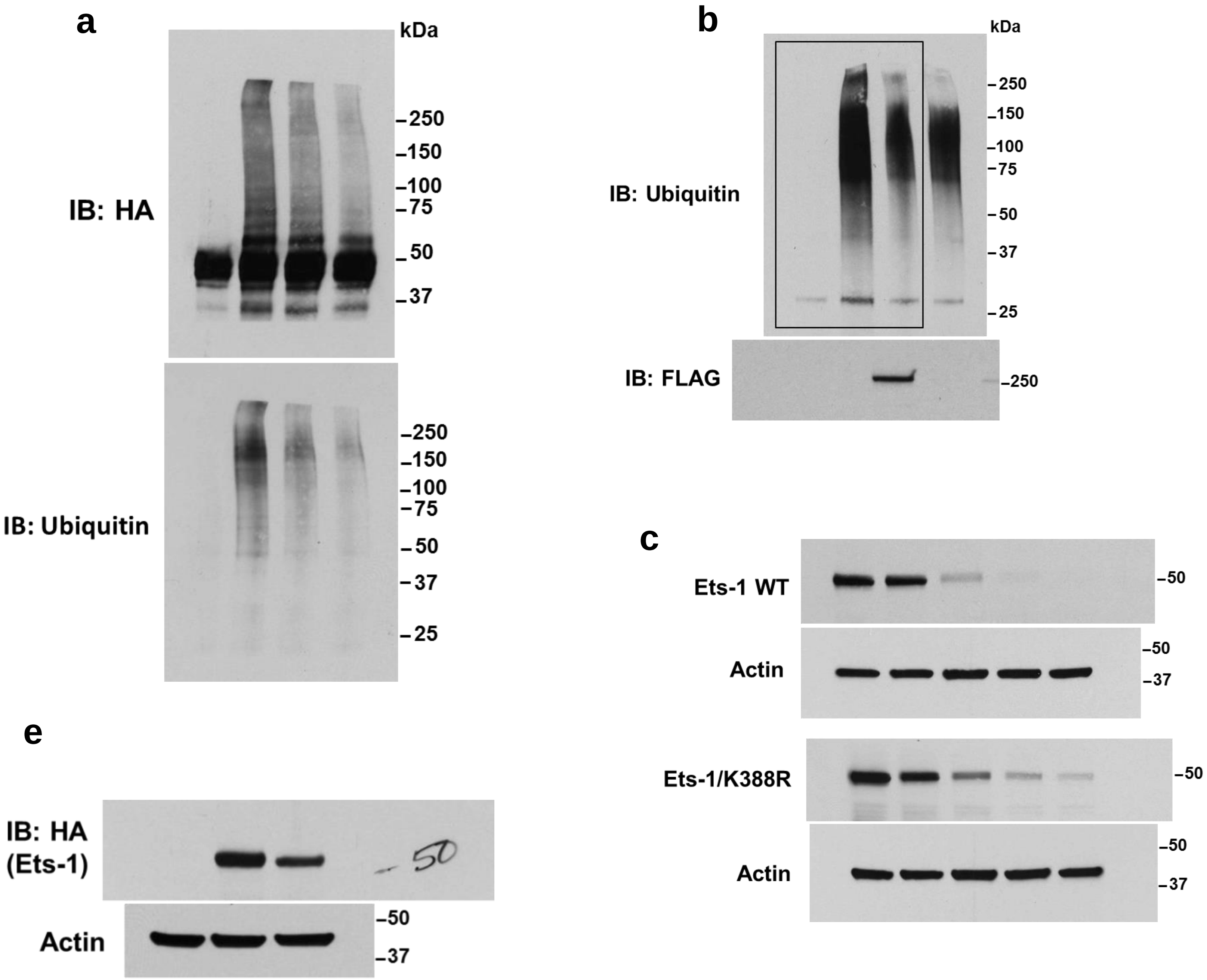

a
IB: HA
kDa
250
150
100
75
50
37
IB: Ubiquitin
250
150
100
75
50
37
25
b
IB: Ubiquitin
kDa
250
150
100
75
50
37
25
IB: FLAG
250
c
Ets-1 WT
50
Actin
50
37
Ets-1/K388R
50
Actin
50
37
e
IB: HA
(Ets-1)
50
Actin
50
37

**Figure 5**

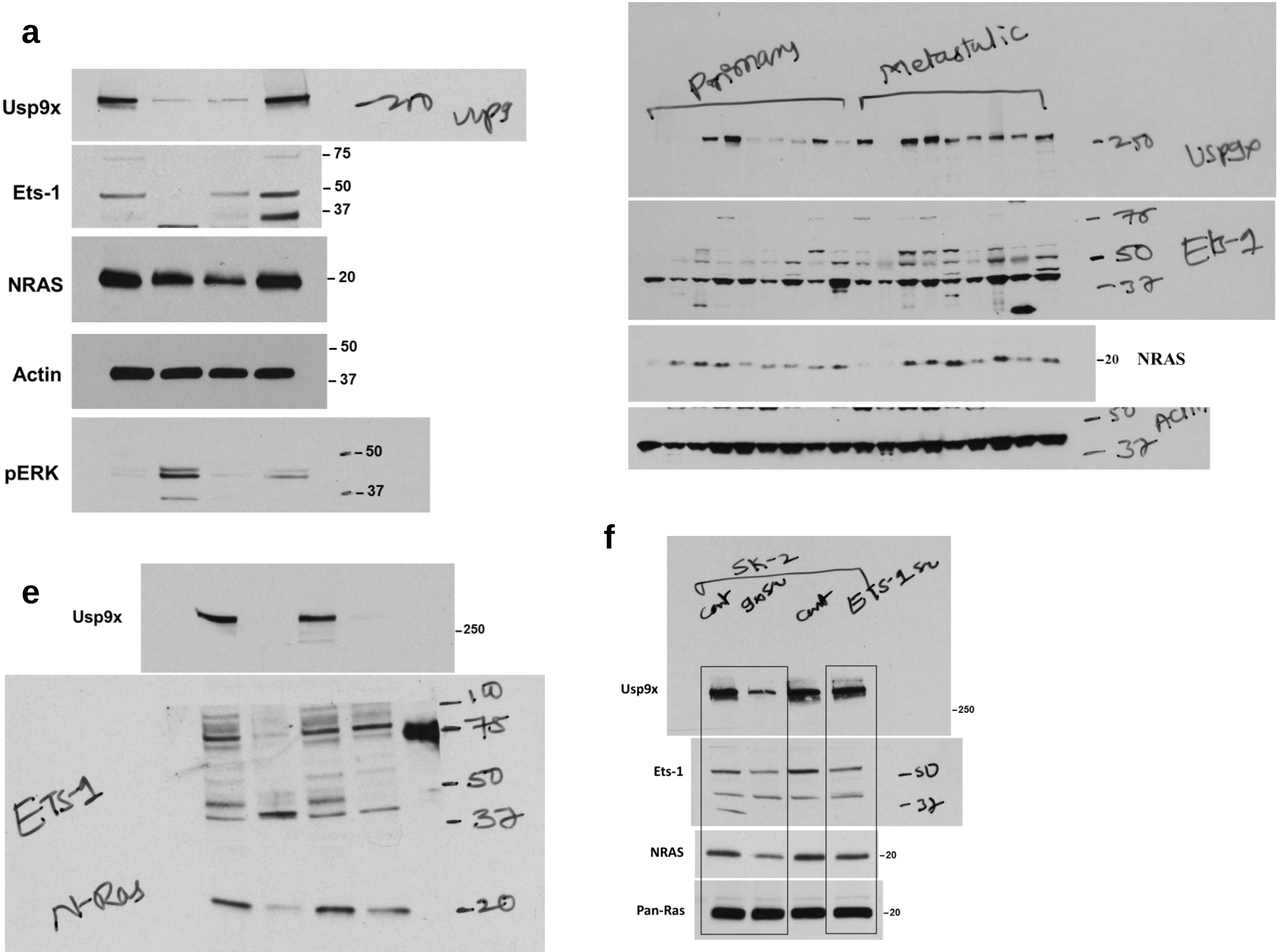

k
a
Usp9x
Ets-1
NRAS
Actin
pERK
75
50
37
20
50
37
50
37
Primary
metastatic
Usp9x
Ets-1
NRAS
-20
e
Usp9x
-250
Ets-1
N-Ras
f
SK-2
Usp9x
Ets-1
NRAS
Pan-Ras
-250
-20
-20

Figure 6

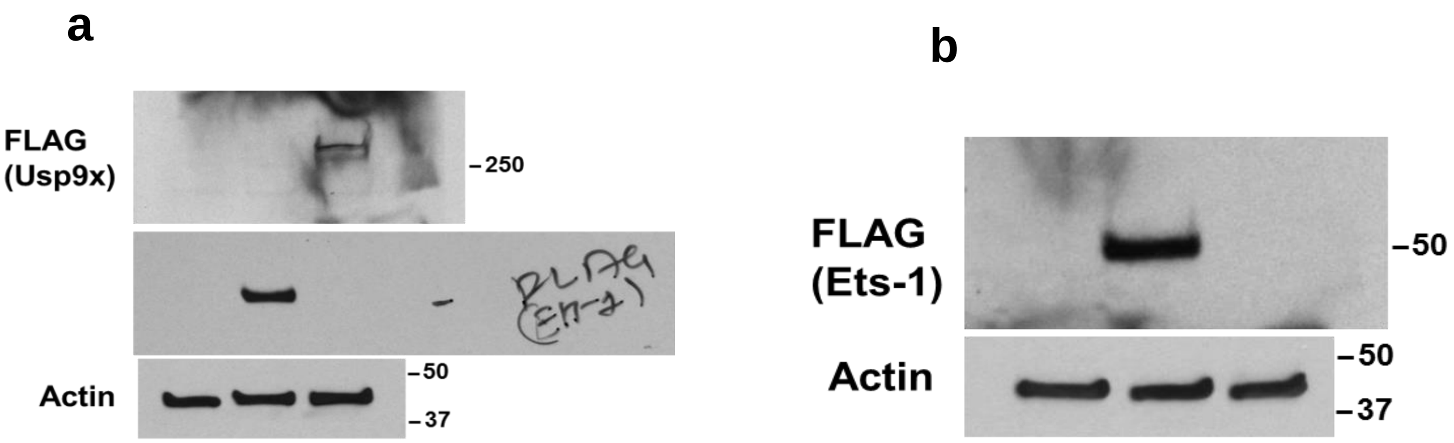

**Figure 7**

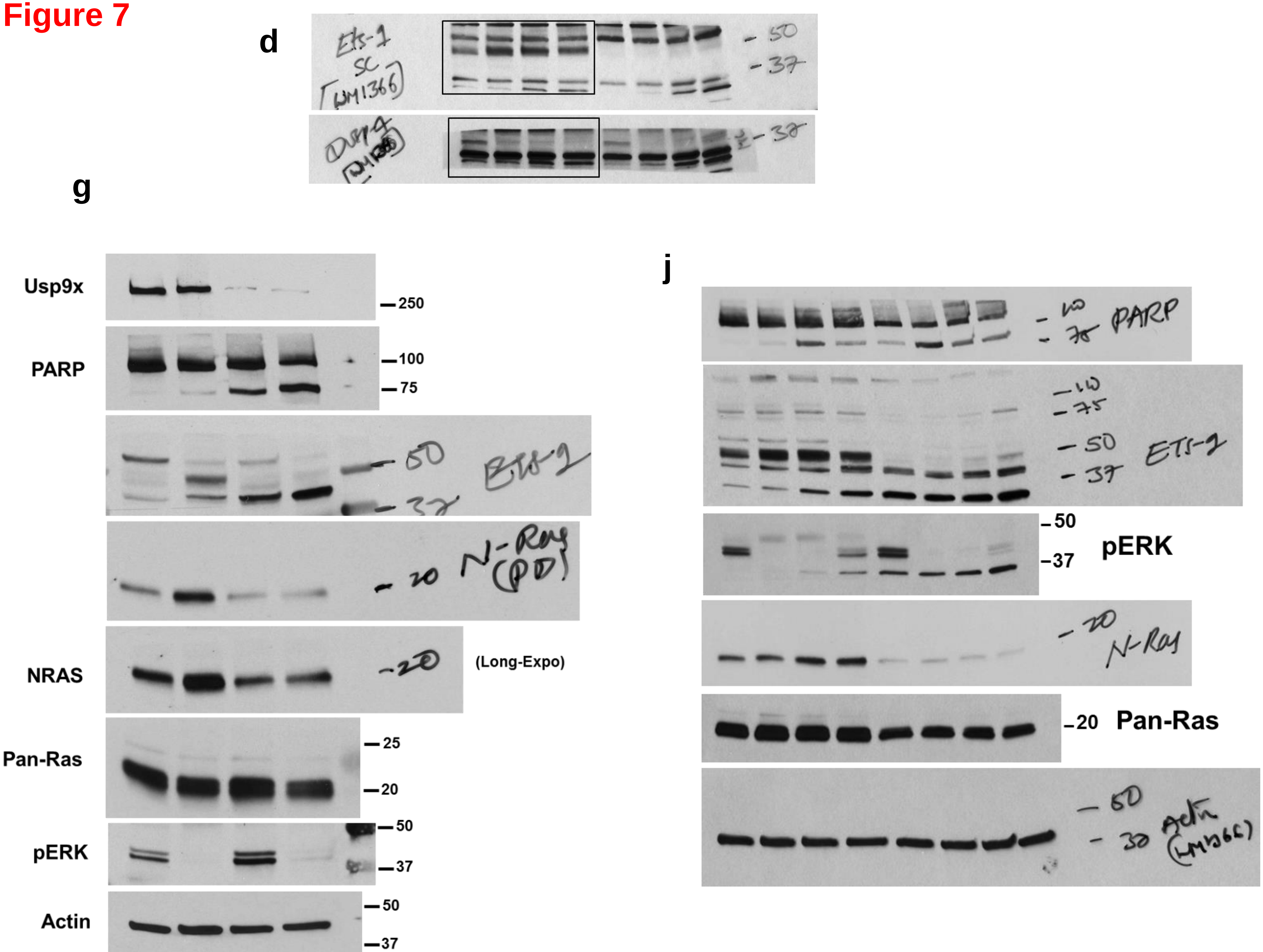

d
g
j
Usp9x
PARP
NRAS
Pan-Ras
pERK
Actin
250
100
75
25
20
50
37
(Long-Expo)
PARP
ETS-1
N-Ras

**Figure 8**

a

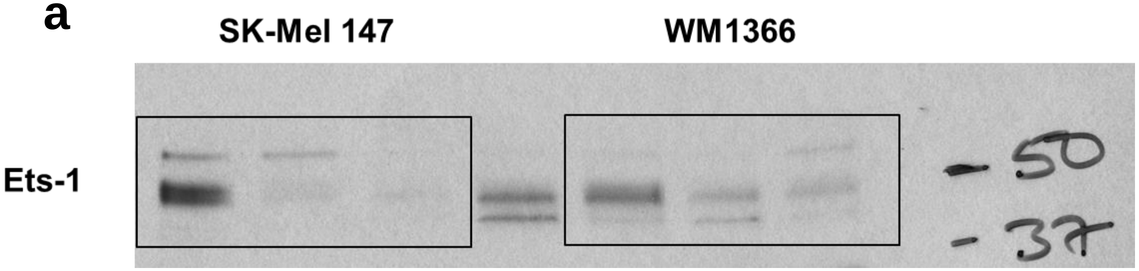


h

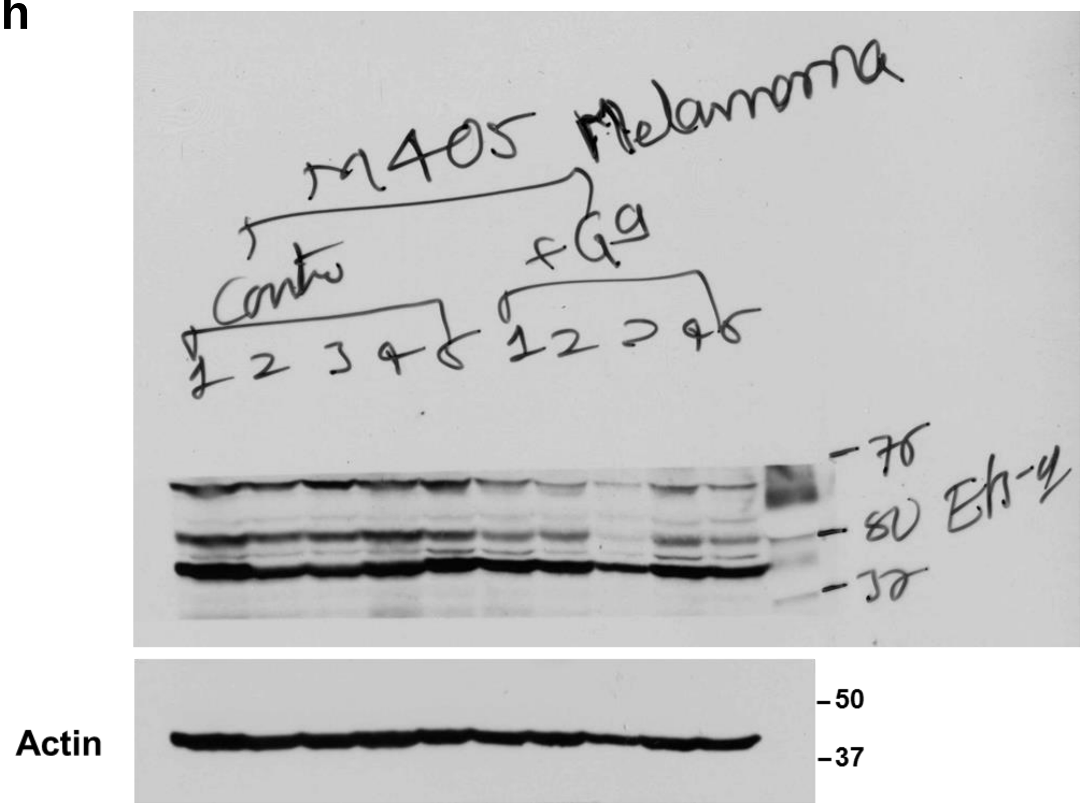

# Supplementary Figure 1

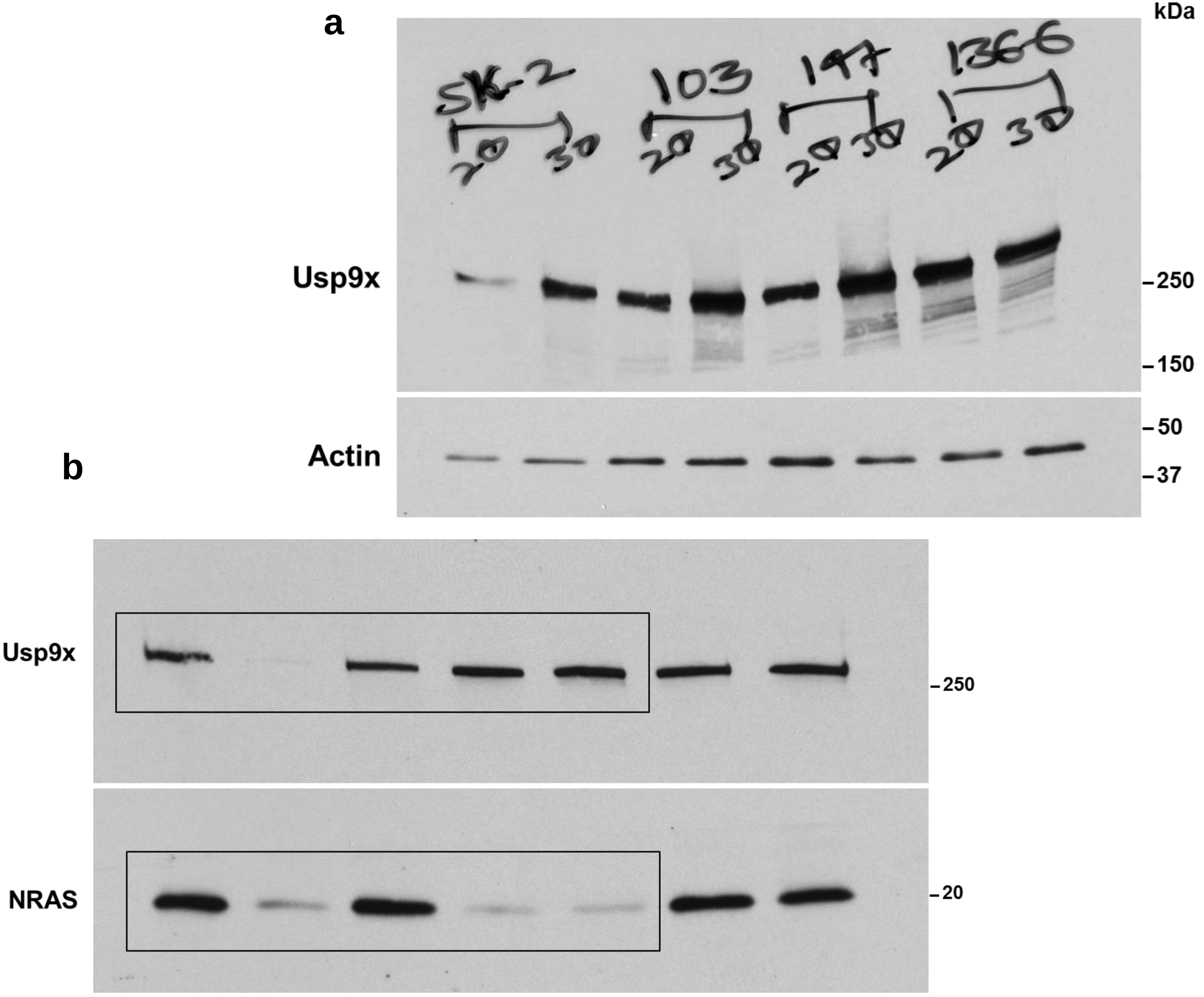

# Supplementary Figure 2

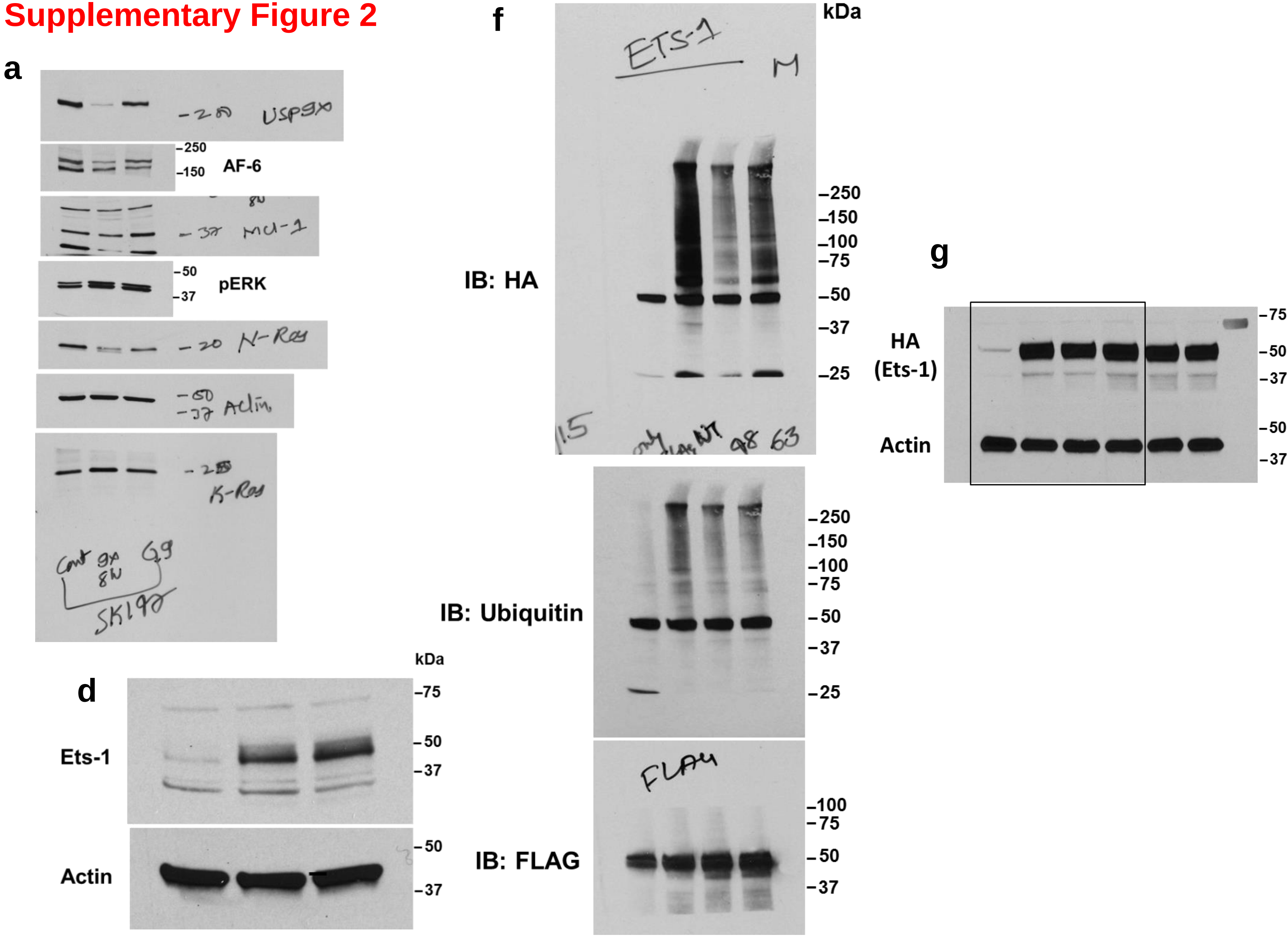

# Supplementary Figure 3

a

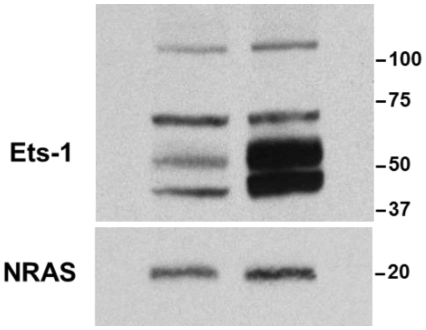


f

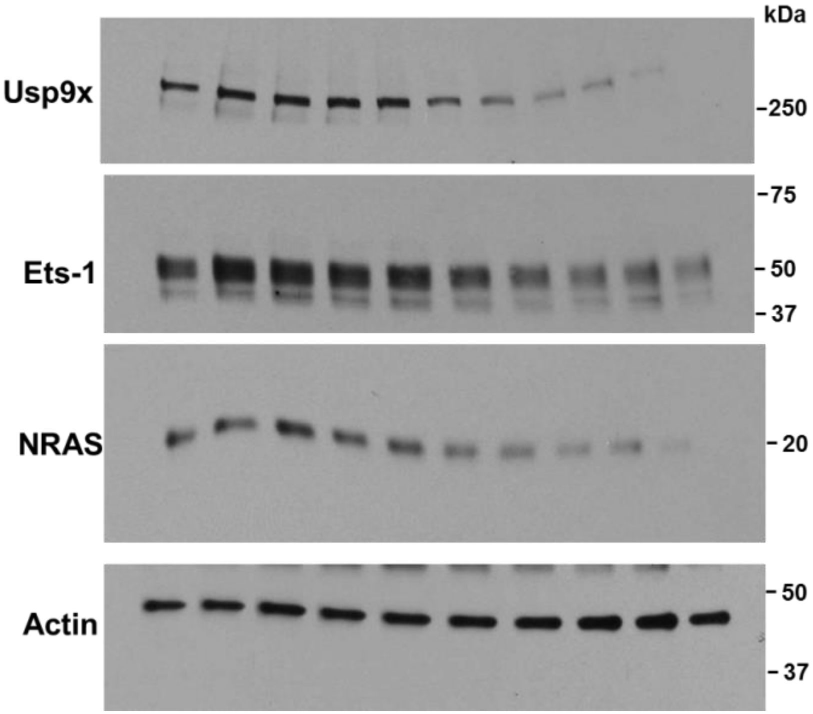


d

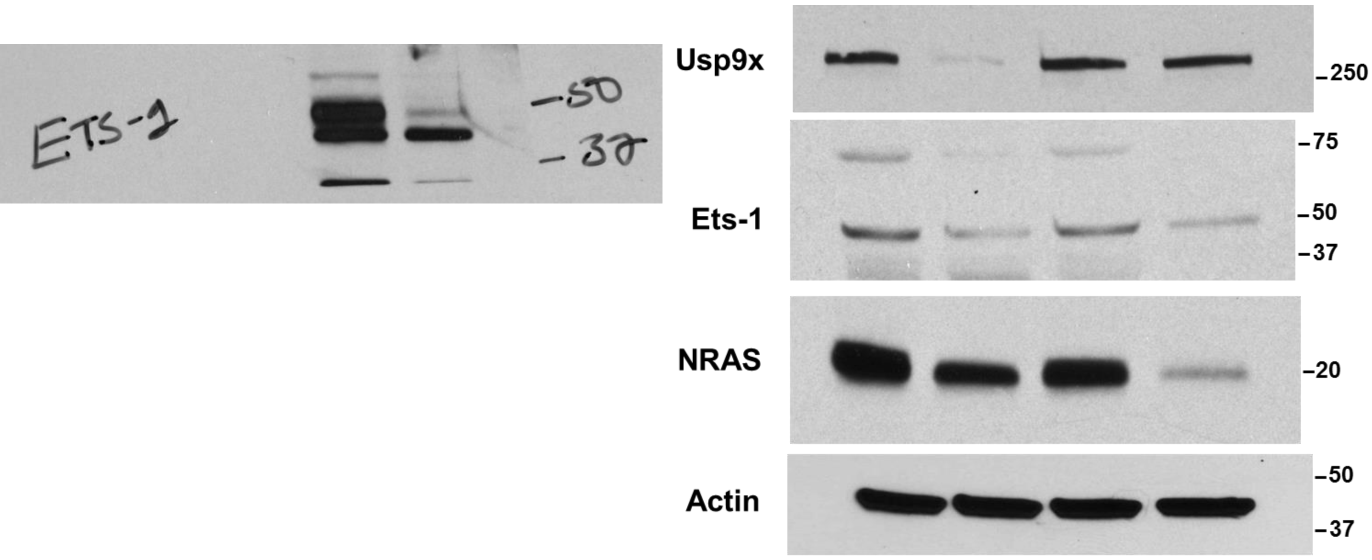


e

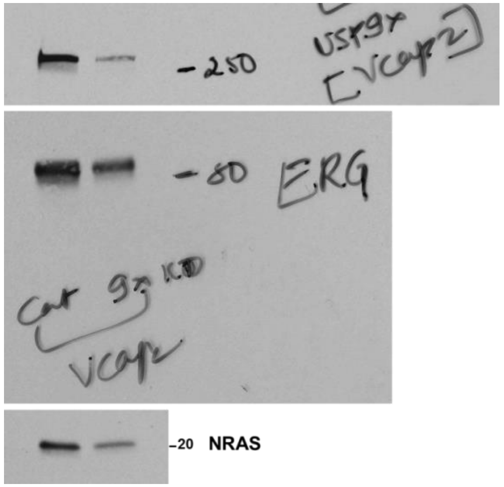

# Supplementary Figure 4

**a**

**Dusp4**

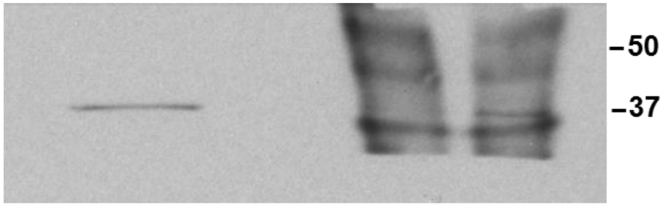


**d**

**Dusp4**

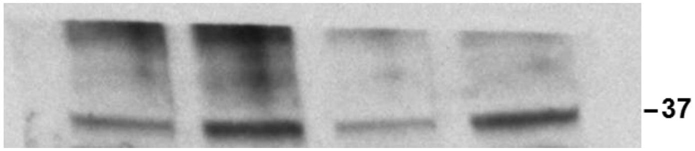


**c**

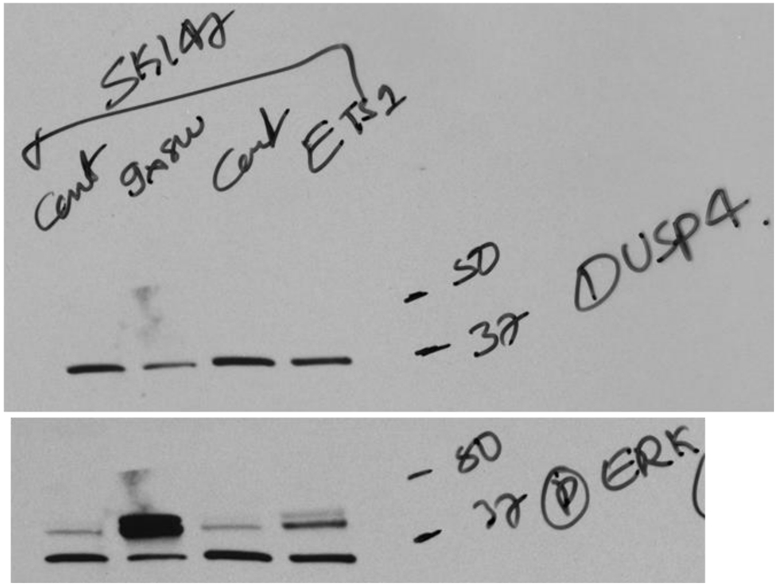

# Supplementary Figure 5

**a**

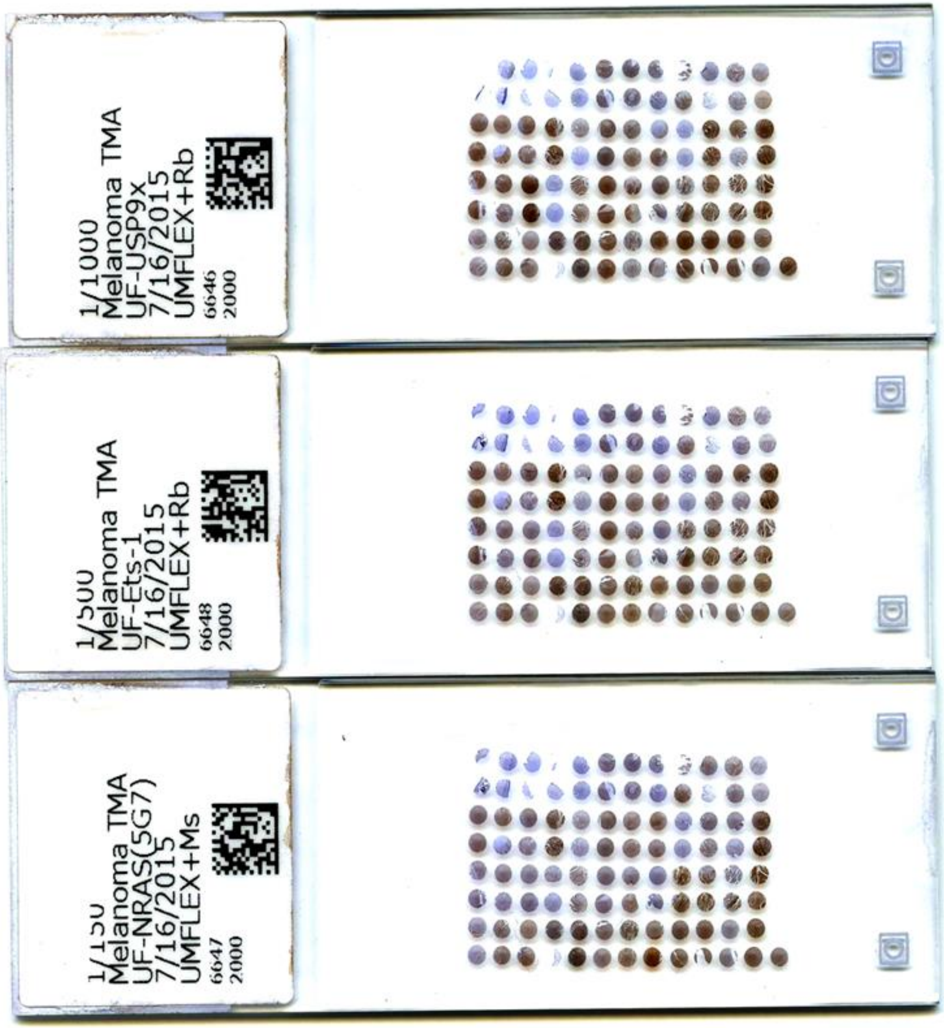


**c**

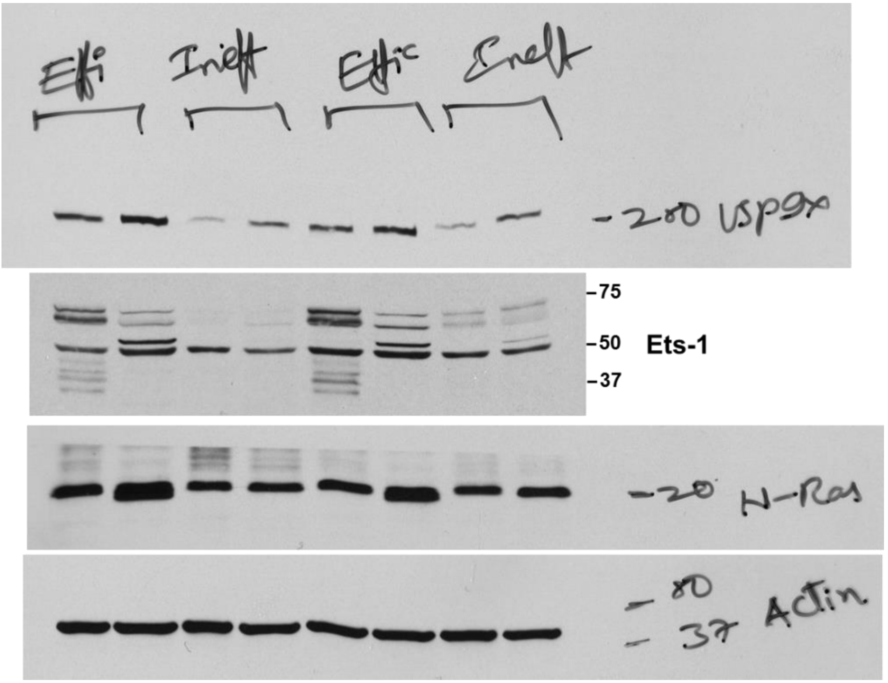

# Supplementary Figure 7

**b**

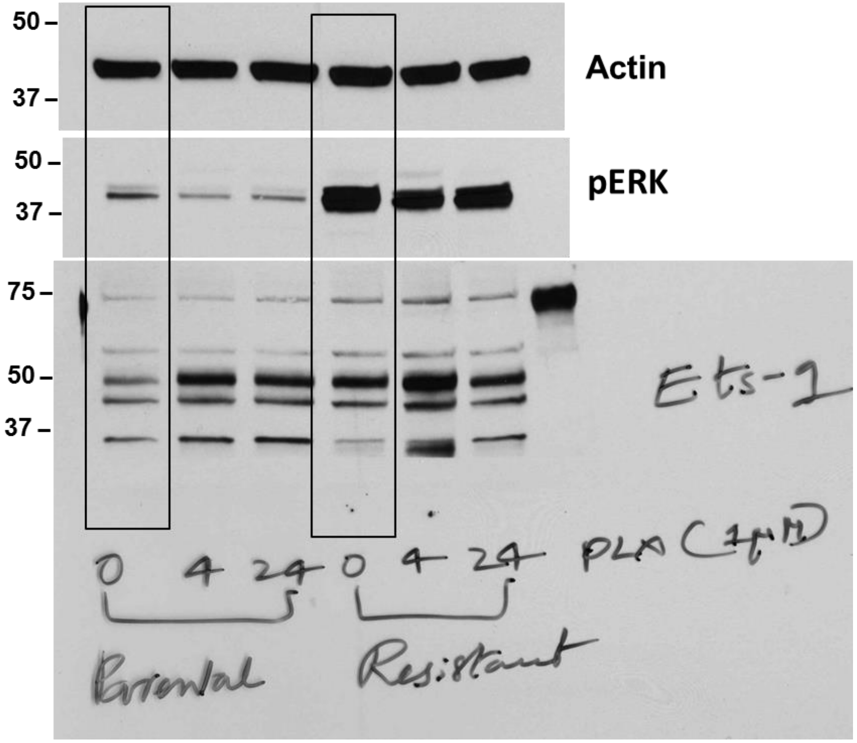


**g**

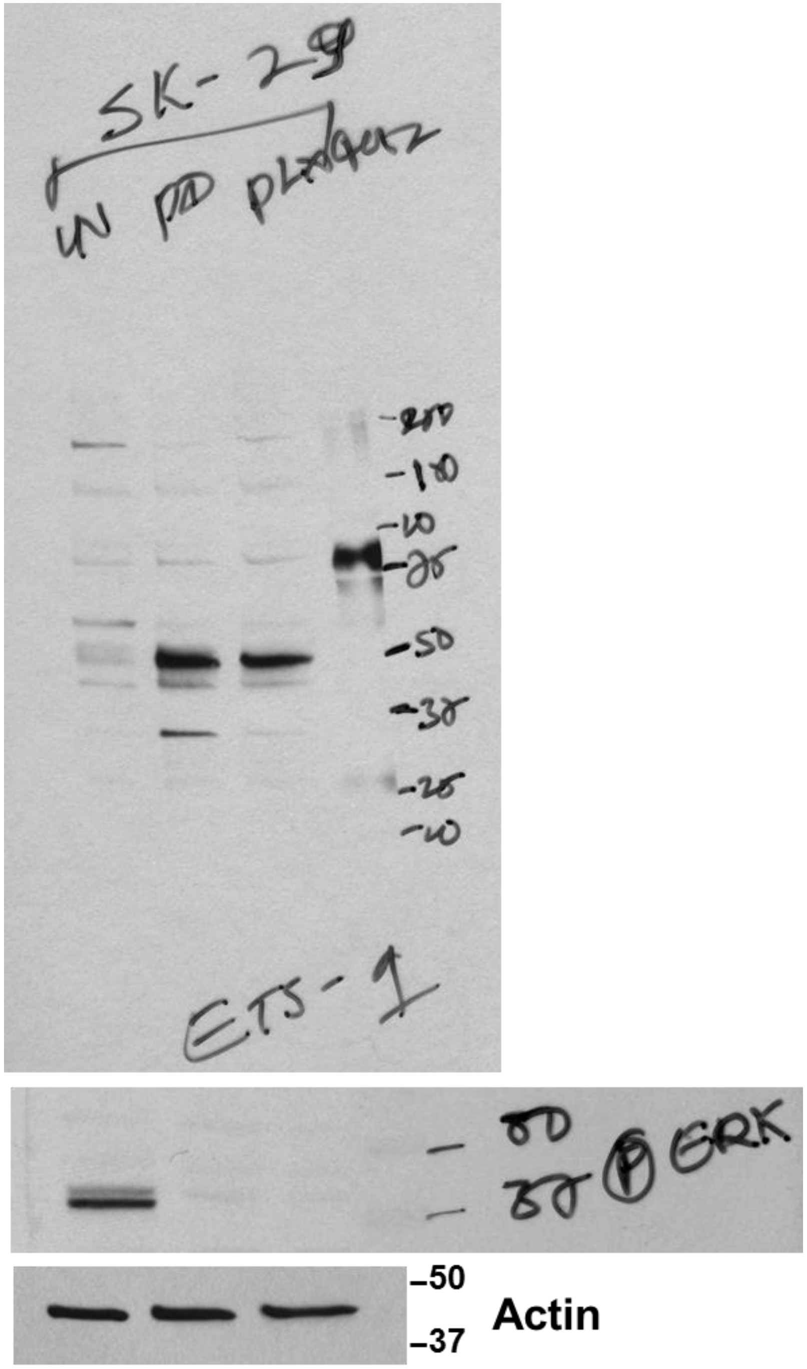

**Supplementary Table 1. Characteristics of melanoma patient tumors analyzed in this study**

| | Tissue# | GENDER | AGE at Tissue Bank | Diagnosis (Breslow depth) | Stage at time of Tissue Bank | BRAF mutation (if known) |
|---|---|---|---|---|---|---|
| Primary | P1 | F | 64 | Primary invasive (3.8 mm) | III | Negative |
| | P2 | M | 62 | Primary ulcerated (8.8 mm) | IIC | |
| | P3 | M | 81 | Primary invasive (3.3 mm) | IIB (T3b N0 M0). | Negative |
| | P4 | F | 68 | Primary invasive (2.2 mm) | IIA (T3a N0 M0). | |
| | P5 | M | 70 | Primary invasive (3.4 mm) | IIC (T4b N0 M0). | |
| | P6 | M | 52 | Ulcerated invasive (6.0 mm) | IIIB | |
| | P7 | M | 62 | Primary melanoma (8 mm) | IIIC (Tx, N3, M0). | Positive |
| | P8 | M | 81 | Primary invasive (9.0 mm) | IIC (T4b N0 M0), | |
| | P9 | M | 87 | Primary invasive (6.40 mm) | IIA (T2b N0 M0) | |
| Metastatic | M1 | M | 60 | Melanoma, probably metastatic (in transit mets) | IIIC (T3b, N2c, M0) | Positive |
| | M2 | M | 64 | Metastatic melanoma involving lymph node with extranodal extension | IIIC (T3b, N1b, M0) | Negative |
| | M3 | M | 48 | Metastatic melanoma | III | Positive |
| | M4 | M | 39 | Metastatic melanoma | IIIC (T3b N1b M0). | Positive |
| | M5 | F | 52 | Metastatic melanoma | IV | Negative |
| | M6 | | 91 | Metastatic melanoma | III | |
| | M7 | M | 76 | Metastatic melanoma | IIIB(Tx, N2b, Mx). | Negative |
| | M8 | M | 57 | Metastatic melanoma | IIIC (T2a N3 M0). | Negative |
| | M9 | M | 86 | Metastatic melanoma | III | |

**Supplementary Table 2. Antibody sources and dilutions used in this study.**

| Protein | Antibody and dilution | Source | Catalogue Number |
|---|---|---|---|
| RAS signaling | Mouse NRAS (1:250) | Santa Cruz | sc-31 (F155) |
| | Mouse Pan-RAS (1:5000) | Santa Cruz | sc-166691 (C-4) |
| | Mouse HRAS (1:250) | Santa Cruz | sc-520 (C-20) |
| | Rabbit Dusp4 (1:1000) | Santa Cruz | sc-1200 (C-18) |
| | Mouse KRAS (1:500) | Calbiochem | OP24 |
| | Rabbit AF-6 (1:1000) | Bethyl Laboratories | A302-199A |
| | Rabbit ERK2 (1:5000) | Santa Cruz | Sc-154 |
| | Rabbit pERK (1:2000) | Cell Signaling | 9101L |
| Ubiquitin signaling | Rabbit Usp9x (1:2000) | Bethyl Laboratories | A301-350A |
| | Mouse Ubiquitin (total) (1:5000) | Santa Cruz | sc-8017 (P4D1) |
| ETS signaling | Rabbit Ets-1 (1:2000) | Bethyl Laboratories | A303-501A |
| | Rabbit ERG (1:4000) | Abcam | ab92513 |
| | Rabbit Ets-2 (1:1000) | Santa Cruz | sc-351 |
| | Rabbit GABPAα (1:1000) | Santa Cruz | sc-22810 |
| | Rabbit Ets-1(C-20) (1:2000) | Santa Cruz | sc-350 |
| Apoptosis signaling | Rabbit Mcl-1(1:1000) | Santa Cruz | sc-819 (S-19) |
| | Rabbit Caspase8 (1:2000) | Cell Signaling | 9746S |
| | Rabbit PARP (1:2000) | Cell Signaling | 9542S |
| | Rabbit BID (1:1000) | Cell Signaling | 2002 |
| | Rabbit BIM (1:1000) | Cell Signaling | 2933T |
| | Rat HA (1:2000) | Roche | 11867423001 |
| | Rabbit HA (Y-11) (1:2000) | Santa Cruz | sc-805 |
| | Mouse FLAG (M2) (1:4000) | Sigma | F1804-1MG |
| | Mouse β-Actin (1:250) | Santa Cruz | sc-69879 (AC-15) |
| Alexa Fluor Conjugated Ab | Alexa Fluor® 488 Goat | Invitrogen | A11034 |
| | Anti-Rabbit IgG (1:5000) | Santa Cruz | sc-2027 |
| Immunohistochemistry | Rabbit Usp9x (1:1000) | Abcam | ab-19879 |
| | Rabbit Ets-1 (1:500) | Bethyl Laboratories | A303-501A |
| | Mouse NRAS (1:150) | Origene | TA505835 (5G7) |

## Supplementary Table 3. Primers used in Ets-1 mutagenesis.

| KPKMNYEKLSRGLRYY (K*388) | Ets-1 Ubiquitin recognition motif |
|---|---|
| 5'-cctaagatgaattatgagaaactgagccgtggcctacgct-3' | **WT** |
| 5'-cctaagatgaattatgagAGActgagccgtggcctacgct-3' (F)<br>5'-agcgtaggccacggctcagTCTctcataattcatcttagg-3' (R) | **Arginine** |
| 5'-cctaagatgaattatgagGCCctgagccgtggcctacgct-3' (F)<br>5'-agcgtaggccacggctcagGGCctcataattcatcttagG-3' (R) | **Alanine** |

## Supplementary Table 4. Primers used in mutation of the putative ETS binding site in the NRAS promoter.

| Primer sequence (5' to 3') | Primer name | N | %GC | %mismatch | Tm |
|---|---|---|---|---|---|
| TTCAACTTTATATCACGAGCATGGATGGGTCTGAT | Ets1E1MF | 35 | 40.00 | 5.7142857 | 72.90 |
| ATCAGACCCATCCATGCTCGTGATATAAAGTTGAA | Ets1E1MR | 35 | 40.00 | 5.7142857 | 72.90 |
| GGAGGGCGGTTTTATCTGCTCCCTTGTCGTTTGAGG | Ets1E2MF | 36 | 55.60 | 5.5555555 | 79.99 |
| CCTCAAACGACAAGGGAGCAGATAAAACCGCCCTCC | Ets1E2MR | 36 | 55.60 | 5.5555555 | 79.99 |
| GTTAATGGCGAAAGAATAGCAGCGAATAAAGTTTTAC | Ets1E3MF | 37 | 35.10 | 5.4054054 | 72.24 |
| GTAAAACTTTATTCGCTGCTATTCTTTCGCCATTAAC | Ets1E3MR | 37 | 35.10 | 5.4054054 | 72.24 |
| TACGTAGCGGGCGGGGCCGAACGTGCCGCTCCTTGGTGGG | Ets1E4MF | 40 | 72.50 | 5 | 89.35 |
| CCCACCAAGGAGCGGCACGTTCGGCCCCGCCCGCTACGTA | Ets1E4MR | 40 | 72.50 | 5 | 89.35 |
| GGGGCTGTTCATGGCGGTGCTGGGGTCTCCAACATTTAAG | Ets1E5MF | 40 | 72.50 | 5 | 89.35 |
| CTTAAATGTTGGAGACCCCAGCACCGCCATGAACAGCCCC | Ets1E5MR | 40 | 72.50 | 5 | 89.35 |

## Supplementary References


1 Quintana, E. *et al.* Human melanoma metastasis in NSG mice correlates with clinical outcome in patients. *Science translational medicine* **4**, 159ra149, doi:10.1126/scitranslmed.3004599 (2012).

2 Liu, F. *et al.* Stat3-targeted therapies overcome the acquired resistance to vemurafenib in melanomas. *The Journal of investigative dermatology* **133**, 2041-2049, doi:10.1038/jid.2013.32 (2013).

3 Pratilas, C. A. *et al.* (V600E)BRAF is associated with disabled feedback inhibition of RAF-MEK signaling and elevated transcriptional output of the pathway. *Proc Natl Acad Sci U S A* **106**, 4519-4524, doi:10.1073/pnas.0900780106 (2009).

4 Obenauf, A. C. *et al.* Therapy-induced tumour secretomes promote resistance and tumour progression. *Nature* **520**, 368-372, doi:10.1038/nature14336 (2015).
